# Identification of large genomic rearrangement of *BRCA1/2* in high risk patients in Korea

**DOI:** 10.1186/s12881-017-0398-3

**Published:** 2017-03-28

**Authors:** Do-Hoon Kim, Hyojin Chae, Irene Jo, Jaeeun Yoo, Hyeyoung Lee, Woori Jang, Joonhong Park, Gun Dong Lee, Dong-Seok Jeon, Keun Ho Lee, Soo Young Hur, Byung Joo Chae, Byung Joo Song, Myungshin Kim, Yonggoo Kim

**Affiliations:** 1 Department of Laboratory Medicine, Seoul St. Mary’s Hospital, College of Medicine, The Catholic University of Korea, 222, Banpo-daero, Seocho-gu, Seoul, 06591 Republic of Korea; 2 Department of Laboratory Medicine, Keimyung University School of Medicine, Daegu, South Korea; 3 Catholic Genetic Laboratory Center, Seoul St. Mary’s Hospital, College of Medicine, The Catholic University of Korea, Seoul, Republic of Korea; 4 Department of Obstetrics and Gynecology, College of Medicine, The Catholic University of Korea, Seoul, Republic of Korea; 5 Department of Surgery, College of Medicine, The Catholic University of Korea, Seoul, Republic of Korea

**Keywords:** BRCA1, BRCA2, Breast cancer, Ovarian cancer, Genetic testing, Korea

## Abstract

**Background:**

While the majority of germline inactivating mutations in *BRCA1/2* are small-scale mutations, large genomic rearrangements (LGRs) are also detected in a variable proportion of patients. However, routine genetic methods are incapable of detecting LGRs, and comprehensive genetic testing algorithm is necessary.

**Methods:**

We performed multiplex ligation-dependent probe amplification assay for small-scale mutation negative patients at high-risk for LGR, based on previously published LGR risk criteria. The inclusion criteria for the high-risk subgroup were personal history of 1) early-onset breast cancer (diagnosed at ≤36 years); 2) two breast primaries; 3) breast cancer diagnosed at any age, with ≥1 close blood relatives (includes first-, second-, or third-degree) with breast and/or epithelial ovarian cancer; 4) both breast and epithelial ovarian cancer diagnosed at any age; and 5) epithelial ovarian cancer with ≥1 close blood relatives with breast and/or epithelial ovarian cancer.

**Results:**

Two LGRs were identified. One was a heterozygous deletion of exon 19 and the other was a heterozygous duplication of exon 4–6. The prevalence of LGRs was 7% among Sanger-negative, high-risk patients, and accounted for 13% of all *BRCA1* mutations and 2% of all patients. Moreover, LGRs reported in Korean patients, including our 2 newly identified cases, were found exclusively in families with at least one high-risk feature.

**Conclusions:**

Our result suggests that selective LGR screening for Sanger-negative, high-risk patients is necessary for Korean patients.

## Background

Breast cancer was the second most common cancer in females aged 15–64 in Korea [1] and in 2013, a total of 17,292 incident breast cancer cases were reported in Korea Central Cancer Registry [2]. Breast cancer incidence in Korea has markedly increased in recent years, and the crude incidence of breast cancer in Korea is the highest among Asian countries [3]. Also, the average age at onset of breast cancer is 10 years earlier than Western populations. A younger age at diagnosis suggests that genetic susceptibility genes may be involved in a substantial proportion of breast cancer in Korea.

Recently, genetic counselling and genetic testing of *BRCA1* and *BRCA2* for the presence of germline inactivating mutations have been increasingly offered to identify individuals at elevated risk of breast and ovarian cancer in Korea. According to a large nationwide prospective Korean Hereditary Breast Cancer (KOHBRA) study, 153 distinct *BRCA1/2* mutations have been identified in Korean breast cancer patients with a family history of breast/ovarian cancer resulting in a prevalence of 22.3% [3].

However, until recently, testing of *BRCA1* and *BRCA2* mutations has been focused on the identification of small-scale mutations (point mutations, small deletions and insertions). Such mutations occur throughout the whole coding sequence and at the splice junctions of both genes. They result in protein truncation, disruption of messenger RNA processing, or amino acid substitutions that have significant impact on protein function and are readily detectable by standard methods of Sanger sequencing of polymerase chain reaction (PCR)-amplified gene segments [4]. Large genomic rearrangement (LGR) of *BRCA1* and *BRCA2*, which is another mechanism of gene inactivation, is responsible for a variable but significant proportion of *BRCA* mutations [5]. High prevalence of LGRs in *BRCA1* have been demonstrated in several populations, including Dutch, Northern Italian, French, and Czech, and in such populations, LGR screening has been advocated as a cost-effective, initial phase screening test [6]. In Korea, reported LGR cases are few, and routine PCR-based genetic testing methods are not capable of detecting LGRs. Therefore, an efficient genetic testing algorithm that incorporates LGR testing is necessary for accurate mutational screening of high-risk patients.

Herein, we performed multiplex ligation-dependent probe amplification (MLPA) for LGR analysis in a subset of small-scale mutation negative patients who were also stratified as high-risk for LGR based on previously published LGR risk criteria from other ethnicities [5, 7]. Here we report the prevalence of the different type of *BRCA* mutations according to risk stratification, to provide evidence for developing an effective and comprehensive *BRCA* genetic screening strategy in Korean patients.

## Methods

### Patients and clinical diagnosis

A total of 106 patients at risk for hereditary breast and ovarian cancer (HBOC) and for whom mutation analysis was requested from January 2015 to November 2015 at Seoul St. Mary’s Hospital were included in this study. The family history, past medical history, and tumor pathology of the probands and their family members were detailed by their referring physicians and/or through review of patient’s medical records. All participants gave informed consent, and this study was approved by the Institutional Review Board (IRB)/Ethics Committee of Seoul St. Mary’s Hospital (IRB No.KC15RISI0915).

The referred patients all met the National Comprehensive Cancer Network genetic testing criteria for HBOC syndrome [8] and high-risk subgroup for LGR was defined based on the previously published LGR risk criteria [5, 7, 9]. The inclusion criteria for the high-risk subgroup were personal history of 1) early-onset breast cancer (diagnosed at ≤36 years); 2) two breast cancer primaries; 3) breast cancer diagnosed at any age, with ≥1 close blood relatives (includes first-, second-, or third-degree) with breast and/or epithelial ovarian cancer; 4) both breast and epithelial ovarian cancer diagnosed at any age; and 5) epithelial ovarian cancer with ≥1 close blood relatives with breast and/or epithelial ovarian cancer.

### Sanger sequencing

Sanger sequencing was performed in all patients to detect small-scale mutations. Genomic DNA was isolated from the peripheral blood leukocytes, using the QIAmp DNA Mini Kit (Qiagen, Hamburg, Germany). Sanger sequencing was performed as described previously [10]. Exon numbering and DNA sequence variant descriptions are based on NM_007294.3 and NM_000059.3 as reference sequences for *BRCA1* and *BRCA2*. To classify variants, we followed the standards and guidelines of the American College of Medical Genetics and Genomics (ACMG) for the interpretation of sequence variants [11], and all variants were scored and classified into five pathogenicity groups (class 1: benign; class 2: likely benign; class 3: uncertain significance (VUS); class 4: likely pathogenic; class 5; pathogenic).

### MLPA analyses

MLPA was performed for all Sanger sequencing-negative patients in the LGR high-risk subgroup. MLPA probe mixes P002 and P045 were used for screening of LGRs in *BRCA1* and *BRCA2*, respectively, and P087 and P077 were used for confirmation, according to the manufacturer’s recommendations (MRC-Holland, Amsterdam, Netherlands). MLPA data were analyzed using Genemarker v1.91 (Softgenetics, State College, PA). Peak heights were normalized and a deletion or duplication was defined as recommended by the manufacturer. Direct sequencing of the probe binding and ligation sites was performed in relevant exons to detect if any polymorphism was located close to the ligation site, which may lead to a false decrease in peak signal [4].

### Statistical analyses

Categorical variables were compared using the Chi-square test. Continuous variables were compared using the independent samples *t*-test or Mann–Whitney-Wilcoxon rank sum tests. MedCalc version 12.1.4 (MedCalc Software, Mariakerke, Belgium) was used and *P* < 0.05 was considered statistically significant.

## Results

### Patient risk characteristics

A total of 106 patients at risk of HBOC were enrolled. All were female patients with a mean age of 51 years and mean age at diagnosis was 48 years. Sixty-six patients (62%) were ovarian cancer cases and 40 patients (38%) were breast cancer cases with 7 having both breast and ovarian cancer (Table 1).

**Table 1:** Baseline characteristics of the study population

Characteristic	Total	Breast cancer	Ovarian cancer
Number	106	40^a^ (38%)	66 (62%)
Age, years			
Mean (SD)	51 (12)	46	54
Age at diagnosis, years			
Mean (SD)	48 (12)	44	51
High-risk features (BC)			
Family history (1st-3rd degree)^d^		19 (48%)^b^	
Early-onset (≤36 years)		11 (28%)^b^	
Bilateral		8 (20%)^b^	
BC + OC		7 (18%)^b^	
Multiple risks		9 (23%)^b^	
≥ 1 high-risk feature		35 (88%)^b^	
High-risk features (OC)			
Family history (1st-3rd degree)^d^			9 (14%)^c^

*BC* breast cancer, *OC* ovarian cancer

^a^Seven patients had both breast and ovarian cancer

^b^The percentage of patients with each high-risk feature among BC patients. Because of rounding, the total does not equal 100%

^c^The percentage of patients with high-risk feature among OC patients

^d^Family history of BC and/or OC in ≥1 close blood relatives (includes first-, second-, or third-degree relatives)

When the patient’s risk of HBOC was stratified according to the aforementioned high-risk criteria, 44 patients (35 breast and 9 ovarian cancer patients) were classified as high-risk (Table 1).

### *BRCA1* and *BRCA2* mutation screening

**Fig. 1:**
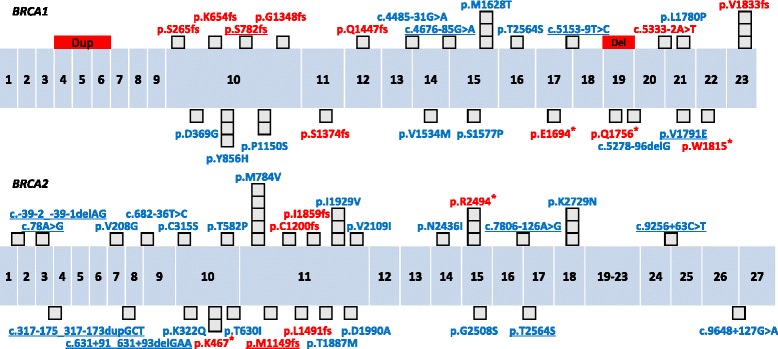
Deleterious mutations (*red labels*) and variants of unknown significance (*blue labels*) of BRCA1/2 genes found in this study. The allele count of each variant is represented by the number of black square and novel variations are underlined. Red squares represent the detected large genomic rearrangements. Distances between variants and dimensions of each exons are not to scale

Of the 106 patients, Sanger sequencing identified 11 different *BRCA1* small-scale mutations in 13 patients (12%) and 6 *BRCA2* small-scale mutations in 9 patients (8%) (Fig. 1). Of the 17 *BRCA1*/*2* small-scale mutations, 1 *BRCA1* (c.2345delG) and 1 *BRCA2* mutations (c.3445_3446dupAT, *n* = 3) were novel, and 1 *BRCA1* (c.5496_5506delGGTGACCCGAGinsA) and 2 *BRCA2* mutations (c.1399A > T, *n* = 2; c.7480C > T, *n* = 3) were recurrent. In addition, 13 different *BRCA1* VUSs and 22 different *BRCA2* VUSs were detected and p.M784V in *BRCA2* was the most common VUS (Fig. 1).

**Fig. 2:**
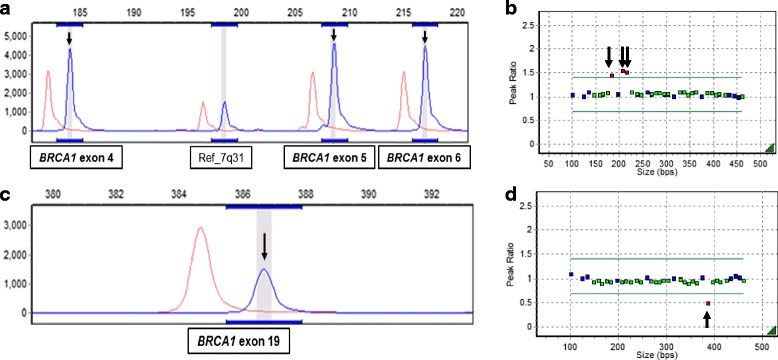
The 2 BRCA1 LGRs identified in the study using MLPA. (**a**, **b**) MLPA analysis of the patient who carries duplication of BRCA1 exon 4-6. (**c**, **d**) MLPA analysis of the patient who carries deletion of BRCA1 exon 19

MLPA analysis of *BRCA1* and *BRCA2* in Sanger-negative, high-risk patients revealed 2 previously reported LGRs: a duplication of *BRCA1* exon 4–6 and a deletion of *BRCA1* exon 19 (Fig. 2). The LGRs consisted 13% of all identified *BRCA1* mutations and the prevalence of LGRs identified in this study was 7% in 29 Sanger-negative, high-risk patients and 2% of all enrolled patients.

### *BRCA1* and *BRCA2* mutation prevalence and risk factors

The overall prevalence of *BRCA* mutations (including both small-scale mutations and LGRs) was 20% of all patients, 30% (12/40) for breast cancer patients, and 18% (12/66) for ovarian cancer patients. Among patients with personal history of breast cancer, *BRCA* mutations were most frequently detected in those with both breast and ovarian cancer (43%), followed by bilateral breast cancer (38%), and positive family history (37%). Of note, relatively low *BRCA* mutation frequency was found in patients with early-onset breast cancer (18%). And among patients with personal history of ovarian cancer, *BRCA* mutations were found in 55% of those patients with the single high-risk criterion of positive family history (Table 2).

**Table 2:** Risk characteristics of patients with small-scale mutations and large genomic rearrangements

Characteristic	mutation negative^a^	mutation positive		*p*-value^b^
		small-scale mutations	large genomic rearrangements	
Number	82	22	2	
Age, years				
Mean (SD)	51 (13)	51 (9)	55 (18)	
Age at diagnosis, years				
Mean (SD)	48 (12)	48 (9)	49 (25)	
BC	28	11	1	
OC	54	11	1	
High-risk features (BC)				
Family history (1st-3rd degree)^c^	12	6	1	0.5804
Early-onset (≤ 36 years)	9	1	1	0.5365
Bilateral	5	2	1	0.9313
BC + OC	4	3		0.7164
Multiple risk	9	4	1	
≥ 1 high-risk feature	24	10	1	
High-risk features (OC)				
Family history (1st-3rd degree)^c^	5	5	1	0.0831
High-risk features (overall)				
≥ 1 high-risk feature	29	15	2	**0.0326**

*BC* breast cancer, *OC* ovarian cancer

Bold numbers represent statistical significance

^a^Both small-scale mutation and large genomic rearrangement negative

^b^For a test of the hypothesis that the frequency of each high-risk feature in mutation negative and mutation positive group is equal

^c^The percentage of patients with high-risk feature among OC patients

Although not statistically significant, each of the high-risk features was more commonly observed in the mutation positive group than in the mutation negative group, except for the high-risk feature of early-onset in breast cancer patients. When the presence of at least one of these high-risk features are compared between the mutation positive group and the mutation negative group, the proportion of patients with at least one high-risk was twice as high in the mutation positive group (71%) than in the mutation negative group (35%), with statistical significance (*P* = 0.0326).

### Risk characteristics of Korean patients with LGR

**Fig. 3:**
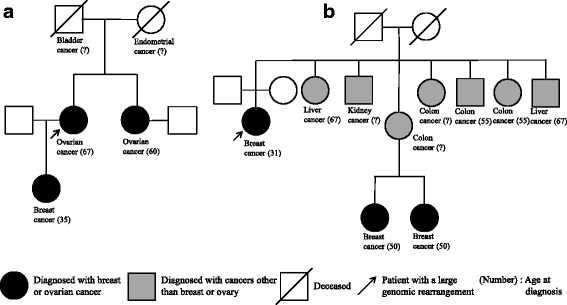
Pedigree of the 2 patients with large genomic rearrangements identified in this study. (**a**) A patient with a duplication of *BRCA1* exon 4-6 and (**b**) a patient who carried a deletion of *BRCA1* exon 19

The two patients identified in this study with LGRs all had at least one high-risk feature (Fig. 3). The index patient with a duplication of *BRCA1* exon 4–6 was diagnosed at the age of 67 with invasive serous epithelial ovarian cancer, had 2 close blood relatives with breast and/or epithelial ovarian cancer and also had 2 close blood relatives with cancers other than breast or ovary. The index patient with a deletion of *BRCA1* exon 19 had a personal history of early-onset, bilateral, triple-negative breast cancer, had 2 close blood relatives with breast cancer and also had 7 close blood relatives with cancers other than breast or ovary.

Also, when mutation probabilities were retrospectively calculated in previously reported Korean LGR probands [12–14] including our 2 newly identified cases by BRCAPRO [15–17] and Korean hereditary breast cancer study *BRCA* risk calculator (KOHCal) [18], 67% of patients presented with BRCAPRO mutation probability >10% and 100% of patients presented with KOHCal mutation probability of >10%. Moreover, all reported LGRs in Korea were detected in patients with at least one high-risk feature (Table 3).

**Table 3:** Risk characteristics of *BRCA1* large genomic rearrangements reported in Korea

Exon rearrangement	primary cancer	Age (at Dx)	FHx	High risk features	BRCAPRO	KOHCal	Detection method	Reference
Duplication of exon 4–6	OC	35	1 BC, 1 OC, 2 COBO	Strong FHx	0.7296	NA	MLPA	This study
Deletions of exon 10–12	BC	35	2 BC	Early-onset BC Strong FHx	0.2635	69.3	MLPA, long PCR	10
Deletions of exon 12–14	BC	35	1 BC, 1 OC	Early-onset BC Strong FHx	0.4352	33.4	MLPA	10
Deletions of exon 12–14	BC	49	2 BC, 1 COBO	Early-onset BC Strong FHx	0.0015	19.0	MLPA, long PCR	9
Deletion of exon 19	BC	31	2 BC, 7 COBO	Early-onset BC Bilateral BC Strong FHx	0.0961	76.9	MLPA	This study
Deletions of exon 1–23	BC	36	4 BC	Strong FHx	0.8132	30.4	MLPA	10
Deletions of exon 1–23	BC	NA	NA	NA	NA	NA	MLPA	1

*BC* breast cancer, *OC* ovarian cancer, *COBO* cancers other than breast or ovary, *Dx* diagnosis, *FHx* family, *MLPA* multiplex ligation-dependent probe amplification, *NA* not available

## Discussion

In this report of 106 consecutive Korean patients at risk for HBOC from a single center cohort, we identified 2 LGRs in Sanger-negative, high-risk patients. Also, 11 *BRCA1* and 6 *BRCA2* small-scale mutations were identified and our report extends the spectrum of *BRCA* mutations by detecting two novel frame-shift mutations in *BRCA1/2*. The overall prevalence of *BRCA* mutations was 20% for all patients, 30% for breast cancer, and 18% for ovarian cancer patients.

The frequency of mutations was related to the type as well as the number of risk factors. Strong predictors of the likelihood of carrying a *BRCA* mutation in patients with personal history of breast cancer were the occurrence of both breast and ovarian cancer (43%), bilateral breast cancer (38%), and positive family history (37%), and the presence of positive family history (55%) in patients with personal history of ovarian cancer. However, there was no difference between the proportion of early-onset breast cancer patients between the mutation positive group and the mutation negative group. Furthermore, all of the *BRCA* mutation-positive patients with early-onset breast cancer, had multiple risk factors other than early-onset. This is in agreement with earlier reports that in Korean, non-familial, early-onset breast cancer patients without other risk factors, the prevalence of *BRCA* mutation is low [3, 18].

The most important finding of this study is that selective screening of high-risk, Sanger-negative Korean patients for LGRs using MLPA analysis, identified 2 patients with LGRs in *BRCA1*. The LGRs comprised 2% of all enrolled patients but accounted for 7% of Sanger-negative, high-risk patients and 12% of all identified *BRCA* mutations in high-risk patients. The frequency of LGR varies considerably among populations, with LGRs accounting for one third of *BRCA1* mutations in northern Italy, and 27–36% of *BRCA1* mutations in Netherlands, but less common in other populations [9]. LGRs in *BRCA* are considered to be rare in Korea, with a reported frequency of 0.45% in familial breast cancer patients [12] and 2.1% in Sanger-negative, familial breast cancer patients [14]. And, only 5 LGR cases have been reported so far in Korea [3]. However, the characterization of risk characteristics of LGRs in any given population allows a more efficient and cost-effective mutational screening approach. Our results suggest that risk stratification and selectively screening Sanger-negative, high-risk patients for LGRs is an effective screening strategy for LGR detection in Korean patients. And with emerging therapies, such as poly ADP ribose polymerase inhibitors in combination with conventional treatment [19], a comprehensive genetic evaluation strategy encompassing LGRs as well as small-scale mutations has become even more critical.

This study has certain limitations. We did not perform MLPA for all Sanger-negative patients, but only in high-risk patients, and the number of LGR cases is limited. And since, Sanger-negative, non-high-risk patients were not included in the MLPA analysis, the true frequency of LGRs may be greater than the reported 2% of all enrolled patients. However, previous reports have indicated that patients harboring LGR appears to be at the highest end of the range of risks associated with *BRCA* mutation [5, 9], and the probability of an LGR among non-high-risk patients can be regarded as significantly low. Whereas the present study is limited by small sample size and was not designed to provide a comprehensive survey of the frequency of LGRs, our data suggest that in high-risk families with a negative Sanger sequencing result, LGRs represent a significant source of *BRCA* mutations.

## Conclusions

In summary, we have applied a simple LGR risk criteria, and have shown that screening Sanger-negative high-risk patients for LGR is an effective and necessary genetic testing strategy in Korean patients. Based on the results of this study, risk stratification of at risk patients for HBOC and selective screening for LGRs in *BRCA1* is recommended for Korean patients.

## Abbreviations

HBOC: Hereditary breast and ovarian cancer
IRB: Institutional review board
KOHBRA: Korean hereditary breast cancer (study)
KOHCal: Korean hereditary breast cancer study BRCA risk calculator
LGR: Large genomic rearrangement
MLPA: Multiplex ligation-dependent probe amplification
PCR: Polymerase chain reaction
